# Antimicrobial Photodynamic Treatment with Mother Juices and Their Single Compounds as Photosensitizers

**DOI:** 10.3390/nu13030710

**Published:** 2021-02-24

**Authors:** Sigrun Chrubasik-Hausmann, Elmar Hellwig, Michael Müller, Ali Al-Ahmad

**Affiliations:** 1 Institute of Forensic Medicine, Faculty of Medicine, University of Freiburg, 79104 Freiburg, Germany; sigrun.chrubasik@klinikum.uni-freiburg.de; 2 Department of Operative Dentistry and Periodontology, Medical Center, Faculty of Medicine, University of Freiburg, 79106 Freiburg, Germany; elmar.hellwig@uniklinik-freiburg.de; 3 Institute of Pharmaceutical Sciences, Pharmaceutical and Medicinal Chemistry, University of Freiburg, 79104 Freiburg, Germany; michael.mueller@pharmazie.uni-freiburg.de

**Keywords:** *Punica granatum*, *Vaccinium myrtillus*, *Aronia melanocarpa*, punicalagin, cyanidin 3-glucoside and hyperoside, *Streptococcus mutans*, *Streptococcus sobrinus*, photosensitizer, antimicrobial photodynamic treatment

## Abstract

The potent antimicrobial effects of antimicrobial photodynamic therapy (aPDT) with visible light plus water-filtered infrared-A irradiation and natural compounds as photosensitizers (PSs) have recently been demonstrated. The aim of this study was to obtain information on the antimicrobial effects of aPDT with mother juices against typical cariogenic oral *Streptococcus* pathogens in their planktonic form and determine its eradication potential on total human salivary bacteria from volunteers. Mother juices of pomegranate, bilberry, and chokeberry at different concentrations were used as PSs. The unweighted (absolute) irradiance was 200 mW cm^−2^, applied five minutes. Planktonic cultures of *Streptococcus mutans* and *Streptococcus sobrinus* and total mixed bacteria from pooled saliva of volunteers were treated with aPDT. Up to more than 5 log_10_ of *S. mutans* and *S. sobrinus* were killed by aPDT with 0.4% and 0.8% pomegranate juice, 3% and 50% chokeberry juice, and 12.5% bilberry juice (both strains). Concentrations of at least 25% (pomegranate) and >50% (chokeberry and bilberry) eradicated the mixed bacteria in saliva samples. This pilot study has shown that pomegranate mother juice is superior to the berry juices as a multicomponent PS for killing pathogenic oral bacteria with aPDT.

## 1. Introduction

Caries and chronic inflammation of the periodontal tissues are related to the accumulation of bacterial plaque. Mechanical methods are insufficient to improve periodontitis and often the administration of antibiotics is required following mechanical debridement. However, due to microbial resistance towards most antibiotics as well as disinfectants such as chlorhexidine digluconate and patient allergies, alternative methods are needed. Antimicrobial photodynamic therapy (aPDT) is one such method, being well studied since its discovery around 1900 [1]. The technique requires a visible light source, a non-toxic photosensitizer (PS), and tissue oxygen. The wavelength of the light source needs to be appropriate to excite the PS to produce radicals (singlet oxygen) and/or reactive oxygen species. The latter cause irreversible damage to bacteria, fungi, and viruses [2]. 

As PSs, tetrapyrrole structures and synthetic dyes are in current use, as well as natural compounds such as the red colors hypericin and riboflavin (vitamin B2), yellow curcumin [1], quercetin (a flavonol) [3], and psoralens [4]. Our group has successfully introduced aPDT with visible light (VIS) plus water-filtered infrared-A (wIRA) irradiation and toluidine blue, chlorine e6 [5,6,7], indocyanine green [8], and hypericum perforatum ethanolic extract [9] as PSs. The results from the aforementioned studies were encouraging for the use of aPDT with VIS + wIRA to treat oral diseases such as periodontitis and peri-implantitis. However, this requires a photosensitizing agent that can be used in the oral cavity without any toxic effects for oral mucous membranes and human cells. Mother juices are freshly squeezed juices from harvested fruit that do not contain any added colorings, preservatives, or sugar. After squeezing, they are pasteurized and immediately bottled. Hence, the aim of this pilot study was to (i) evaluate the phototoxic effects of aPDT with mother juices against typical cariogenic oral *Streptococcus* pathogens in their planktonic form and (ii) determine its eradication potential on total human salivary bacteria from volunteers.

## 2. Materials and Methods

### 2.1. Radiation Source and Photosensitizers

A broadband visible light (VIS) and water-filtered infrared-A (wIRA) radiator (Hydrosun 750 FS, Hydrosun Medizintechnik GmbH, Müllheim, Germany) containing a 7 mm water cuvette was used as described elsewhere in detail [6,9,10]. The radiator comprised a halogen lamp with an accessory water filter (dimensions: length: 28 cm, width: 27 cm, height: 28 cm) using 750 W (voltage: 230 V, 50–60 Hz), which was inserted into the light path for absorbing the infrared-B and -C wavelengths. Additionally, the radiator also included an orange filter (BTE 31, 10 cm in diameter and 200 mW/cm^2^ efficiency). More than doubled-weighted efficient integral illumination with reference to the absorption spectrum of protoporphyrin IX was enabled by using a BTE 31 filter. The 944 and 1180 nm absorption bands were also filtered to prevent superficial overheating. The water-filtered absorption revealed a total spectrum in the range of 570 to 1400 nm, with local minima at around 970, 1200, and 1430 nm. The amount of unweighted radiation applied to the microbial samples was 48 mW cm^−2^ VIS and 152 mW cm^−2^ wIRA (200 mW cm^−2^ in total). All samples were irradiated for 5 min. 

As PSs, we used mother juices of pomegranate (*Punica granatum*, L6349), chokeberry (*Aronia melanocarpa*, L6294), and bilberry (*Vaccinium myrtillus*, L7074) from Biotta AG CH-8274 Tägerwilen, with expiry dates of 7 June 2018, 13 April 2019, and 6 September 2018, respectively (Table 1). The absorbance spectra of all juices were measured using a Tecan Infinite 200 reader (Tecan, Crailsheim, Germany).

In addition, we investigated the compounds punicalagin (PHL80524, Sigma Aldrich, Traufkirchen, Germany), cyanidin 3-glucoside (PHL89616, Merck, Darmstadt, Germany), and hyperoside (0018-05-85, Planegg/Martinsried, Germany). 

### 2.2. Bacterial Strains and Total Human Salivary Bacteria

Two bacterial strains belonging to the mutans streptococci that have been considered as main oral cariogenic bacteria were tested in this study: *Streptococcus mutans* DSM20523, and *Streptococcus sobrinus* DSM 203815. Long-term storage of both bacterial strains was at −80 °C in brain heart infusion containing 15% (*v/v*) glycerol as described by Jones et al. [11] and Al-Ahmad et al. [12]. Maintenance with weekly subculturing was conducted on Columbia blood agar (CBA) at 37 °C in an aerobic atmosphere including 5–10% CO_2_. Overnight cultures of the bacterial strains were prepared in tryptic soy broth (TSB, Merck, Darmstadt, Germany) at 37 °C in an aerobic atmosphere including 5–10% CO_2_. 

Three healthy volunteers gave written consent after the ethics committee of the Albert-Ludwigs-University of Freiburg had approved the study protocol (Nr. 91/13). Exclusion criteria included the use of antibacterial mouth rinses or antibiotics in the three-month period prior to the start of the experiment, pregnancy or lactation, smoking, severe systemic diseases, or participation in another clinical study during the previous three months. The volunteers donated unstimulated human saliva, which was pooled to gain total salivary bacteria. All total salivary samples were taken on the same day, directly before conducting the experiments, to avoid alteration of the total salivary microbiota.

### 2.3. Application Protocol of aPDT 

The bacterial samples and pooled human salivary bacteria were incubated for two minutes in different concentrations of the mother juices prior to irradiation. All dilutions of the mother juices were conducted in 0.95% NaCl solution. The samples were then irradiated by VIS + wIRA for five minutes at 37 °C. Samples treated with chlorhexidine (CHX) served as a positive control. Samples treated with 0.95% NaCl solution, with the mother juices, or solely with irradiation were used as negative controls. The negative controls were incubated in the dark to exclude any accessory effects of day light. The colony-forming units (CFUs) of treated and untreated bacterial strains were determined on CBA at 37 °C and with 5–10% CO_2_ atmosphere for two days and depicted as log10 values. 

Human salivary samples were vortexed for 30 s before determining the CFUs on CBA aerobically at 37 °C and with 5–10% CO_2_ atmosphere for five days. The killing rate in terms of CFU reduction after aPDT was calculated by comparison with the negative control. A statistical analysis was not required if a reduction rate higher than two log10 steps was achieved.

The activity of the PSs remained stable for six months of storage at low temperature (3 °C) in the dark using a light-tight bottle. 

## 3. Results

**Figure 1:**
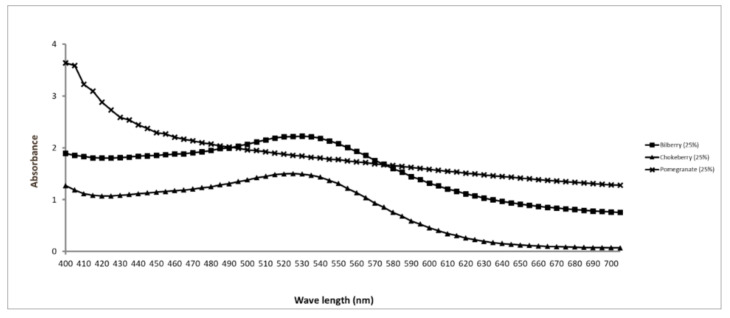
Absorbance spectrum of the mother juices employed in this study.

All three mother juices showed light absorption at a wide wavelength range (Figure 1) close to the emission spectrum of the light source [13].

**Figure 2:**
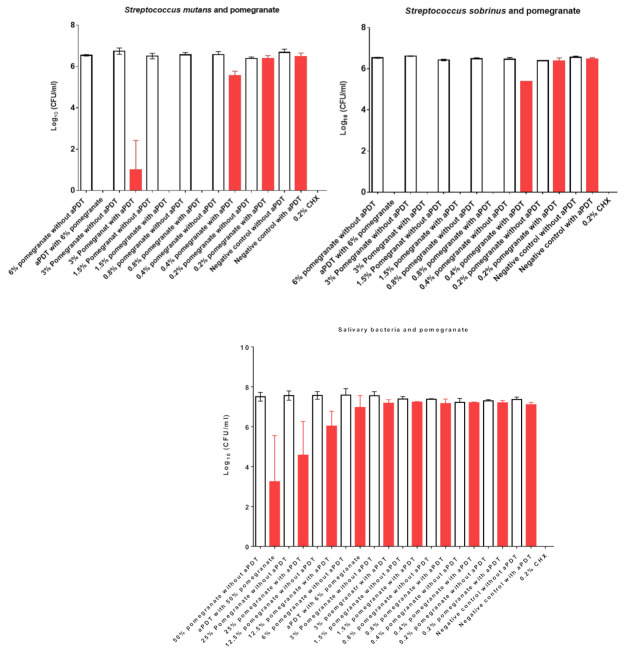
The effects of antimicrobial photodynamic therapy (aPDT) using visible light and water-filtered infrared-A (VIS + wIRA) in combination with pomegranate mother juice as a photosensitizer (PS) against *S. mutans*, *S. sobrinus*, and total salivary bacteria. Untreated and mother juice-treated negative controls are added. Additionally, aPDT without mother juices was conducted as a negative control. Treatment with 0.2% CHX served as a positive control. Red bars depict values with photodynamic treatment; white bars represent values without photodynamic treatment. CHX, chlorhexidine.

The high killing effects of aPDT with pomegranate mother juice on *S. mutans* and *S. sobrinus* are shown in Figure 2 and Table 2. Approximately all bacterial cells were eradicated at PS concentrations ranging between 6% and 0.8%. Even the low concentration of 0.4% showed a high killing rate of one Log_10_ on the planktonic culture of both species. No killing effect of aPDT was shown with 0.2% pomegranate mother juice. The aPDT effects of pomegranate juice on total salivary bacteria (Figure 2) revealed an eradication range between one (12.5%) and 4 Log_10_ (50%). A pomegranate juice concentration lower than 6% showed no antimicrobial effects as a PS with aPDT. The treatment of both pathogens as well as total salivary bacteria with CHX resulted in a total killing effect of 100%.

**Figure 3:**
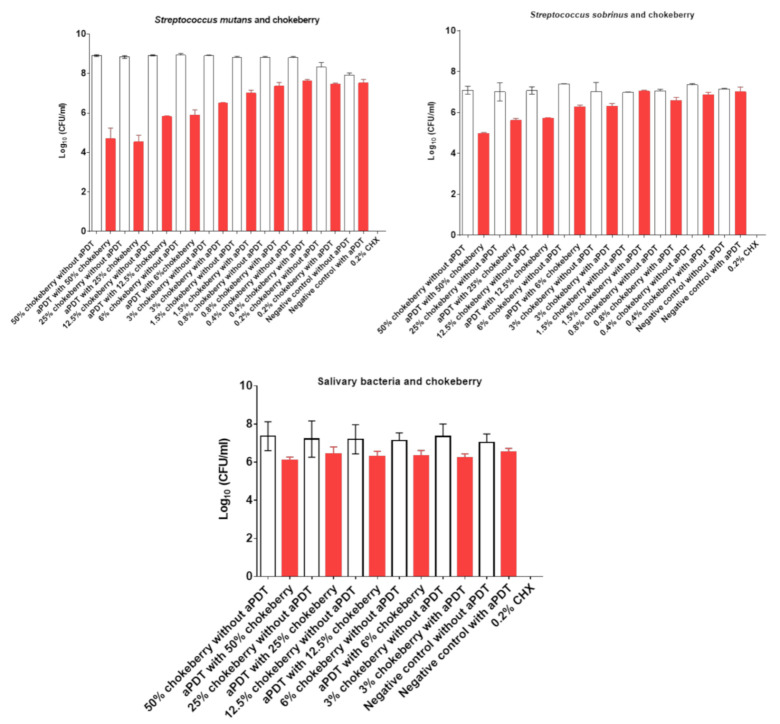
The effects of aPDT using VIS + wIRA in combination with chokeberry mother juice as a PS against *S. mutans*, *S. sobrinus*, and total salivary bacteria. Untreated and mother juice-treated negative controls are added. Additionally, aPDT without mother juices was conducted as a negative control. Treatment with 0.2% CHX served as a positive control. Red bars depict values with photodynamic treatment; white bars represent values without photodynamic treatment. CHX, chlorhexidine; aPDT, antimicrobial photodynamic therapy.

The killing effect of aPDT with chokeberry mother juice on S. mutans (Figure 3, Table 3) was dependent on the concentration of the PS and it ranged from 0.8 (0.2% chokeberry juice) to 4.2 Log10 (50% chokeberry juice). Even the low concentration of 3% showed a high killing rate of 2.4 Log_10_. Similar concentration-dependent antimicrobial effects were shown for *S. sobrinus* (Figure 3, Table 3). The killing of *S. sobrinus* ranged from 0.4 to 2.1 Log_10_ with chokeberry juice concentrations of 0.4% and 50%, respectively. The 6% chokeberry juice showed a killing effect of 1.1 Log_10_ on planktonic *S. sobrinus*. 

The aPDT effects of chokeberry juice on total salivary bacteria (Figure 3, Table 3) revealed an eradication range between 0.8 and 1.3 Log10. The treatment of both pathogens as well as the salivary bacteria with CHX resulted in a total killing effect of 100%.

**Figure 4:**
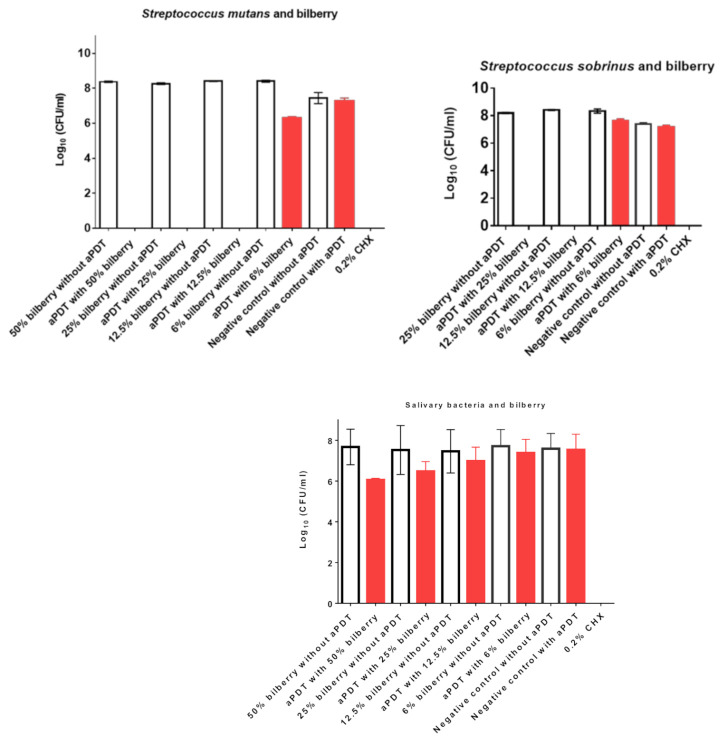
The effects of aPDT using VIS + wIRA in combination with bilberry mother juice as a PS against *S. mutans*, *S. sobrinus*, and total human salivary bacteria. Untreated and mother juice-treated negative controls are added. Additionally, aPDT without mother juices was conducted as a negative control. Treatment with 0.2% CHX served as a positive control. Red bars depict values with photodynamic treatment; white bars represent values without photodynamic treatment. CHX, chlorhexidine; aPDT, antimicrobial photodynamic therapy.

The aPDT with 50%, 25%, and 12.5% bilberry mother juices on *S. mutans* resulted in total killing of all bacteria (Figure 4, Table 4). The aPDT with 6% bilberry juice killed *S. mutans* by two Log_10_ (99%). All negative controls including treatment with pure bilberry juice without irradiation (8.3–8.4 Log10) as well as bacteria incubated solely in 0.95% NaCl solution (with and without irradiation; 7.2–7.4 Log_10_) revealed no antimicrobial effects. The combination of aPDT with 25% and 12.5% bilberry juices also killed all *S. sobrinus* pathogens, whereas the 6% juice reduced the CFUs from 8.3 to 7.6 Log_10_. Treatment with pure juice without irradiation showed no antimicrobial effects (Figure 4, Table 4). Treatment with 0.2% CHX killed all planktonic cells of *S. mutans* and *S. sobrinus* as well as all salivary bacteria. The aPDT with 50% bilberry juice reduced the CFUs of total salivary bacteria from 7.7 to 6.0 Log_10_ (Figure 4, Table 4), reflecting a high killing rate of >90%. A killing effect of one and 0.5 Log_10_ was shown for aPDT with 25% and 12.5% bilberry juices, respectively.

**Figure 5:**
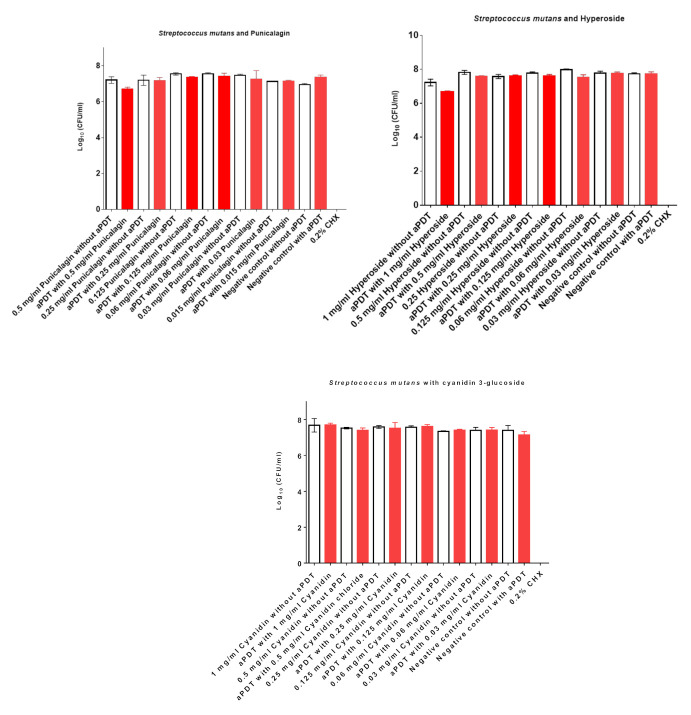
The effects of aPDT using VIS + wIRA in combination with single compounds as PSs against *S. mutans*. Untreated and mother juice-treated negative controls are added. Additionally, aPDT without mother juices was conducted as a negative control. Treatment with 0.2% CHX served as a positive control. Red bars depict values with photodynamic treatment; white bars represent values without photodynamic treatment. CHX, chlorhexidine; aPDT, antimicrobial photodynamic therapy.

**Figure 6:**
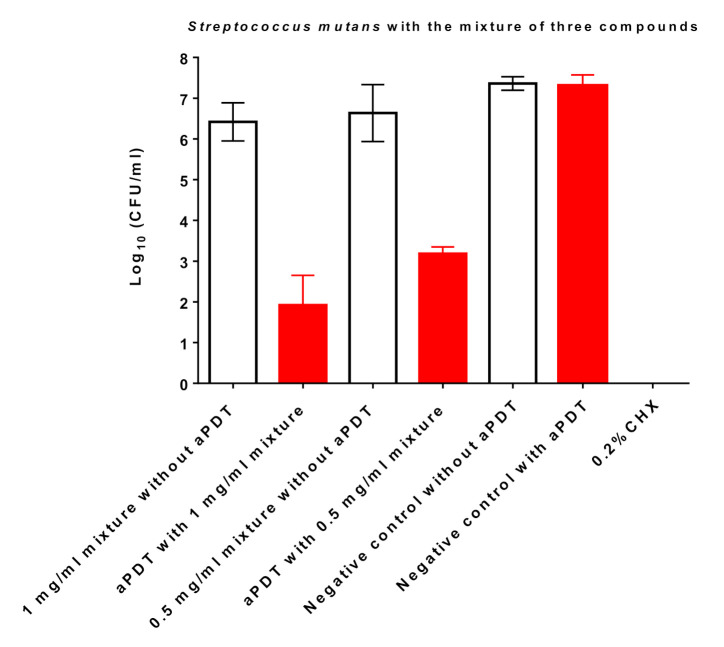
The effects of aPDT using VIS + wIRA in combination with a mixture of all three substances (equal amount of each compound) as a PS against S. mutans. Untreated and mother juice-treated negative controls are added. Additionally, aPDT without a PS was conducted as an additional negative control. Treatment with 0.2% CHX served as a positive control. Red bars depict values with photodynamic treatment; white bars represent values without photodynamic treatment. CHX, chlorhexidine; aPDT, antimicrobial photodynamic therapy.

The aPDT with 0.5 mg/mL punicalgin and 1 mg/mL hyperoside showed only a minor killing effect on *S. mutans* at a range less than 0.5 Log_10_ (Figure 5, Table 5). All other concentrations demonstrated no antimicrobial effect. The aPDT with cyanidin 3-glucoside chloride did not show any killing effects on *S. mutans* at the concentration range of 1 to 0.03 mg/mL. However, the mixture of the three compounds (equal amount of each compound) had a high photosensitizing potential and killed *S. mutans* by more than 4 log_10_ at a concentration of 1 mg/mL (Figure 6). Even at 0.5 mg/mL, the mixture killed *S. mutans* at a bactericidal level of more than 3 log_10_. Treatment only with juices or single compounds without radiation or radiation with VIS + wIRA without PSs showed no antimicrobial effects.

## 4. Discussion

The antimicrobial effects of aPDT with VIS + wIRA in combination with mother juices demonstrated that all juices had a photosensitizing potential that could be used to regain oral health in the case of infections. There are three types of PSs: (i) those that stay in close proximity to a bacterial cell wall, (ii) those that bind to the bacterial cell, which may limit the oxidative damage to outer cell structures, and (iii) those that enter bacterial cells and reach the cytoplasm, which results in damage to intracellular components such as cytoplasmic proteins or DNA [14]. Mother juices per se have a potent antimicrobial activity, although when diluted in non-antimicrobial concentrations, they may serve as PSs. 

### 4.1. Pomegranate

A lipophilic extract of *Punica granatum* was antimicrobial to *S. mutans*, *Staphylococcus aureus*, and *Candida albicans* at bactericidal concentrations of 12.5, 25, and 50 mg/mL, respectively [15]. *S. mutans* was more sensitive to pomegranate than the other microorga-nisms including *Porphyromonas gingivalis* [16]. A hydroalcoholic extract of pomegranate juice inhibited the *S. mutans* Clarke ATCC^®^ 25175™ strain (minimum inhibitory concentration (MIC) 25 μg/μL, minimum bactericidal concentration (MBC) 40 μg/μL) and a *Rothia dentocariosa* clinical isolate (MIC 20 μg/μL, MBC 140 μg/μL) [17]. Pomegranate fruit peel crude extract killed *S. mutans* (MBC 6.25 mg/mL). At sub-bactericidal concentrations, it reduced acid production, biofilm formation, and insoluble extracellular polysaccharide production (EPS) in the biofilm of the planktonic cells of *S. mutans*. The production of soluble EPS was not affected [18].

The antimicrobial activity was attributed to the presence of tannin derivatives including punicalagin [19]. As the mechanism of action, inhibition of extracellular microbial enzymes, deprivation of the substrates required for microbial growth, direct action on microbial metabolism through inhibition of oxidative phosphorylation, and iron deprivation have been suggested [20]. However, other compounds such as phloretin, punigratane, and coutaric acid occurring in trace amounts in the pomegranate showed even higher microbicide effects and may contribute to the overall antibacterial effect [21]. A gel equivalent to 0.234% punicalagin inhibited *S. mutans* and *Streptococcus sanguinis* but not *Lactobacillus casei* within 24 h of incubation in vitro [22]. 

Topalovic and co-workers [23] identified in pomegranate 97 phenolic compounds, 23 anthocyanins and derivatives, 33 ellagitannins and derivatives of ellagic acid, 12 flavanols, 4 flavonol glycosides (among them quercetin, myricetin, kaempferol [24], and hyperoside [25]), one flavone (according to Zhao et al. [24], two flavones: apigenin and luteolin), 17 hydroxybenzoic acids, and 7 hydroxycinnamic acids and derivatives thereof. Flavanols, ellagitannins, and derivatives of ellagic acid had the highest concentrations in pomegranate juice. While isolated colorless tannins were ineffective as PSs for aPDT in killing *S. mutans* (Figure 5, data for procyanidin A2 not shown), the mixture of characteristic compounds contributes to the photosensitizing effect (Figure 6).

Our results show that pomegranate mother juice in concentrations of 0.8% (*S. mutans*) and 0.4% (*S. sobrinus*) with aPDT was associated with a high killing rate of the streptococci. For a high eradication rate of total human salivary samples, a concentration of at least 25% was required. Such concentrations had no impact on the streptococci without aPDT. 

The content of anthocyanins in pomegranate juice of industrial production was on average 1 mg/100 cm^3^, and cyanidin 3,5-*O*-diglucoside accounted for about 40% of them [26], while other studies identified cyanidin 3-*O*-glucoside as the predominant anthocyanin (41%), followed by cyanidin 3,5-*O*-diglucoside (27%) [27]. Cyanidin 3-*O*-glucoside was more instable at the storage temperature than delphinidin 3,5-di- and cyanidin 3,5-diglucosides [28]. Our data show that cyanidin 3-*O*-glucoside in combination with punicalagin and hyperoside may contribute to the PS activity (Figure 6), as quercetin certainly did [3]. It remains to be established whether dilutions of the cytotoxic pomegranate extracts prepared from the whole fruit, juice, or peel with the solvents propylene glycol, ethanol, and methanol, respectively, are also useful PSs. 

### 4.2. Chokeberry

*Aronia melanocarpa* demonstrated potent antimicrobial activity against ten human pathogens [29]. Denev and coworkers investigated crude extract standardized on 20% and 40% anthocyanins on proanthocyanins (PACs), as well as pure compounds (chlorogenic acid, cyanidin 3-*O*-galactoside, epicatechin, rutin, and quercetin). They demonstrated that the antimicrobial effect of chokeberry is mainly due to the action of condensed tannins (PACs), and that quercetin and epicatechin contribute to the antimicrobial activity. By contrast, the anthocyanin fraction was ineffective.

Exposure to 1/10 diluted chokeberry juice for one minute significantly reduced *S. mutans* biofilm formation in vitro without affecting streptococcal growth [30]. One week of oral rinse with diluted chokeberry juice led to significantly fewer salivary streptococcal CFUs than rinsing with water. Lee and coworkers concluded that the juice might inhibit initial biofilm formation by decomposing extracellular RNA [30]. When chokeberry extracts, subfractions, and compounds were tested for their potential to prevent biofilm formation and inhibit bacterial growth of *Escherichia coli* and *Bacillus cereus*, the 50% ethanolic extract was more potent than other extracts. Moreover, epicatechin was the most effective among the compounds tested, while the effects of oligomeric and polymeric PACs were negligible. Interestingly, cyanidin 3-xyloside showed activity against Gram-negative *E. coli* and Gram-positive *B. cereus*, whereas the other anthocyanins were inactive [31].

Chokeberry dry fruit contains up to 7849 mg polyphenols per 100 g. The dark color of the fruit is caused by the high concentration of anthocyanins, which include cyanidin 3-glucoside, 3-galactoside, 3-xyloside, and 3-arabinoside. A small proportion of anthocyanins was attributed to pelargonidine 3-galactoside and 3-arabinoside. Chokeberry flavonols mainly comprise quercetin derivatives and, to a lower degree, isorhamnetin 3-derivatives, myricetin, and kaempherol 3 derivatives. The flavan-3-ols comprise monomeric epicatechin and oligomeric and polymeric PACs. Chlorogenic and neochlorogenic acids are the main phenolic acids [32]. 

Our results indicate that chokeberry mother juice was less effective in killing *S. mutans* and *S. sobrinus* compared with pomegranate mother juice and required concentrations of 6% and 50%, respectively (Figure 3). The killing rate of the mixed bacteria from salivary samples was similar to that of bilberry juice with a concentration of 50% (Figure 3). It remains to be established whether chokeberry extracts are more effective PSs.

### 4.3. Bilberry

The MIC of a lipophilic bilberry (*Vaccinium myrtillus*) extract against *P. gingivalis* was 500 μg/mL [33]. A fraction of it showed antibacterial activity against other oral bacteria with MICs against *P. gingivalis*, *Fusobacterium nucleatum*, and *Prevotella intermedia* of 26, 59, and 45 μg/mL, respectively. The MIC against *S. mutans* was >62.5 μg/mL. It seemed likely that the antimicrobial effect was attributed to the predominant non-anthocyanin phenolic compounds, accounting for approximately 80% of the total phenolic content [34].

Bilberry contains at least 42 bioactive substances including 22 phenolic acids, 15 flavonols, and 5 flavan-3-ols [34]. Derivatives of quercetin, myricetin, isorhamnetin, and kaempferol are among the flavonols identified [35]. The major anthocyanin among the fifteen identified is delphinidin 3-*O*-β-D-glucopyranoside [36]. The group of Liu [37] suggested using cyanidin 3-glucoside as an appropriate marker for bilberry. However, when using several authentic anthocyanin references to quantify anthocyanin contents, a higher anthocyanidin content was revealed, accounting for 1610 mg/L in bilberry juice compared with 417 mg/L in blueberry juice [38]. Thus, anthocyanins predominate in *V. myrtillus*, whereas *Vaccinium angustifolium* contains more hydroxycinnamic acids. The contents of PACs and flavonols are rather similar [39].

Miari and colleagues [40] observed that 25% *V. angustifolium* aqueous extract reduced biofilm formation without affecting the growth of *Pseudomonas aeruginosa*. The decrease in the relative gene expression of exopolysaccharides and quorum sensing encoding genes sheds light on the mechanism of action [40]. Highbush blueberry PACs reduced the growth of *Aggregatibacter actinomycetemcomitans* and prevented biofilm formation at subinhibitory concentrations. PAC treatment of pre-formed biofilms resulted in a loss of bacterial viability, probably due to damage of the bacterial cell membrane. In addition, the PACs protected the oral keratinocytes barrier integrity from damage caused by *A. actinomycetemcomitans* [41]. The same extract with 17% phenolic acids, 13% flavonoids (flavonols, anthocyanins, flavan-3-ols), and almost 3% procyanidins (oligomeric (epi)catechin derivatives) showed antibacterial activity against periodontopathogenic *F. nucleatum* (MIC 1 mg/mL). The authors supposed that the antibacterial activity was caused by the ability of blueberry polyphenols to chelate iron [42]. Blueberry juice also inhibited enzymes such as α-glucosidase dipeptidyl peptidase-4 and tyrosinase dose-dependently. Increased acidity in the biofilms may thus contribute to the antibacterial activity of blueberry preparations [43]. The aforementioned blueberry extract at 62.5 μg/mL inhibited *F. nucleatum* biofilm formation by almost 90%, as well as a number of proinflammatory and cartilage-destructing cytokines (NF-κB, IL-1β, TNF-α, IL-6, MMP-8, and MMP-9). This indicates that blueberry and bilberry extracts have a dual antibacterial and anti-inflammatory action [42]. The latter action is supported by an animal experiment in which a daily oral bilberry extract had a protective effect on oral mucosal damage induced by 5-fluorouracil in hamsters [44], as well as a clinical study in individuals with gingivitis in which the volunteers consumed either 250 or 500 g of bilberries over seven days. The mean reduction in bleeding on probing after consumption of the berries was 41% (250 g) and 59% (500 g) compared with 31% in the placebo group, and 58% in the standard care reference group. Only in the group consuming 500 g of bilberries/day were gingival crevicular fluid cytokines (IL-1ß, IL-6, VEGF) reduced [45].

Our results show that bilberry mother juice is less effective than pomegranate juice as a PS for aPDT. Concentrations of 12.5% and higher were needed to kill the two streptococci species and a concentration of 50% was required to kill total mixed bacteria of pooled human saliva samples. It remains to be established whether bilberry extracts are more effective PSs.

### 4.4. Multicomponent PSs for aPDT 

Our data with isolated compounds indicate that co-administration of an anthocyanin pigment, a flavone (hyperoside), and a tannin (punicalagin) had a photosensitizing effect, although the single compounds had little effect or were ineffective. The flavonol quercetin contained in all mother juices has already been shown to be an appropriate PS [3]. We therefore suggest that the multicomponent cocktail of anthocyanins, flavonols, and tannins contributes to the photosensitizing activity of mother juices. The unique composition of compounds in pomegranate makes this fruit juice superior to berry mother juices. The question of whether to rinse one’s mouth with pomegranate mother juice or its anthocyanin-flavonol-tannin fraction prior to aPDT with VIS + wIRA requires further investigations. To date, the evidence between mother juice intake and damaging caries and tooth erosion is not conclusive. Overall, prospective cohort studies in children and adolescents have found no association between the juice intake and tooth erosion or dental caries, although data from randomized controlled trials (RCT) in adults suggests that the intake of mother juices could worsen oral health. However, the RCT data were from small, short-term studies that utilized intra-oral devices generally devoid of normal plaque or saliva action, and they generally employed conditions that were not reflective of normal juice consumption [46]. 

As oral bacteria exist in the oral biofilm and not in the planktonic state, additional experiments on oral biofilms are required to evaluate the efficiency of this novel aPDT. However, it is important to note that the total salivary bacteria also include flocs of the oral biofilm as has previously been shown by our research group [47]. Hence, the results of the present study give preliminary indications regarding an effect of the applied aPDT on the oral biofilm, which has to be confirmed in future studies.

The lower activity of aPDT against total human salivary bacteria as compared to the effects on *S. mutans* and *S. sobrinus* can be caused by the high heterogeneity of the salivary microbiome that consists of hundreds of different species [48]. More than 700 different species belonging to the Gram-negative and Gram-positive bacteria have been reported as belonging to the oral microbiota [49]. The different structure of the cell wall may lead to a lower sensitivity of Gram-negative bacteria, as the permeability of the photosensitizer could be decreased when compared to the Gram-positive species. Moreover, flocs of oral biofilms have been shown in unstimulated human saliva [47]. Due to the extracellular matrices, these flocs could be less sensitive to the aPDT used as when compared to the planktonic single species cultures of *S. mutans* and *S. sobrinus*.

## 5. Conclusions

This study has revealed a higher killing effect of pomegranate mother juice in low concentration in comparison with bilberry or chokeberry mother juice. Testing of the single compounds showed that only a mixture of different components has a photosensitizing killing effect in combination with VIS + wIRA. Due to the known healing effects of wIRA on human cells, the aPDT using VIS + wIRA in combination with components of pomegranate fruit juice may be a promising technique for treating oral infections. In order to evaluate the potential of this, novel aPDT future clinical studies should be conducted using different fractions of pomegranate mother juice.

## Figures and Tables

**Table 1:** Polyphenol content (Folin–Ciocalteu method, mg/100 mL) of the mother juices. The ana-lyses were carried out by Pharma Bellavista GmbH, CH 9404 Rorschacherberg, using established methods defined in the European Pharmacopeia.

	Pomegranate	Bilberry	Chokeberry
Total polyphenols calculated as pyrogallols *	384	693	697
Tannins calculated as pyrogallols *	218	371	446
Procyanidins calculated as cyanidin chloride **	100		836
Ellagic acid ***	8.6		

* PhEur 2.8.14, ** PhEur 2.2.25, *** PhEur 2.2.29.

**Table 2:** Killing effects of aPDT using pomegranate mother juice as a photosensitizer.

Target	Concentration of the Photosensitizer	Killing Effects
*S. mutans*	0.8–6%	≥3 Log10
	0.2–0.4%	≤1 Log10
*S. sobrinus*	0.8–6%	≥3 Log10
	0.2–0.4%	≤1 Log10
Total salivary bacteria	25–50%	≥3 Log10
	12.5%	≤1 Log10

**Table 3:** Killing effects of aPDT using chokeberry mother juice as a photosensitizer.

Target	Concentration of the Photosensitizer	Killing Effects
*S. mutans*	6–50%	≥3 Log10
	1.5–3%	≤2.4 Log10
	0.2–0.8%	≤1 Log10
*S. sobrinus*	50%	2.1 Log10
	6–25%	1–1.4 Log10
Total salivary bacteria	50%	1.3 Log10
	0.3–25%	≤0.8 Log10

**Table 4:** Killing effects of aPDT using bilberry mother juice as a photosensitizer.

Target	Concentration of the Photosensitizer	Killing Effects
*S. mutans*	12.5–50%	≥3 Log10
	6%	2 Log10
	0.2–0.8%	≤1 Log10
*S. sobrinus*	12.5–25%	≥3 Log10
	6%	0.9 Log10
Total salivary bacteria	50%	1.7 Log10
	25%	1 Log10
	12.5%	0.5 Log10

**Table 5:** Effects of aPDT using single compounds as photosensitizers against *Streptococcus mutans*.

Compound	Concentration of the Photosensitizer	Killing Effects
Punicalagin	0.015–0.5 mg/mL	0.4 Log10
	0.015–0.5 mg/mL	no effects
Hyperoside	1 mg/mL	0.5 Log10
	0.03–0.5 mg/mL	no effects
Cyanidin 3-glucoside chloride	0.03–1 mg/mL	no effects

## Data Availability

Data available on request due to restrictions e.g., privacy or ethical. The data presented in this study are available on request from the corresponding author.
